# Dangerous crossing: demographic and clinical features of rescued sea migrants seen in 2014 at an outpatient clinic at Augusta Harbor, Italy

**DOI:** 10.1186/s13031-016-0080-y

**Published:** 2016-06-15

**Authors:** Alessia Trovato, Anthony Reid, Kudakwashe C. Takarinda, Chiara Montaldo, Tom Decroo, Philip Owiti, Francesco Bongiorno, Stefano Di Carlo

**Affiliations:** Medici Senza Frontiere Italia, Via Magenta 5, Roma, 00185 Italy; FCFP, Médecins Sans Frontières Bruxelles, Operational Research Unit Luxembourg, 68 rue de Gasperich L-1617, Luxembourg City, Luxembourg; International Union Against Tuberculosis and Lung Disease (IUATLD), Ministry of Health & Child Care, AIDS & TB Department, Cnr 5th Street & Livingstone Avenue, Harare, Zimbabwe; Médecins Sans Frontières Bruxelles, Operational Centre Brussels, Medical Department, Operational Research Unit Luxembourg, 68 rue de Gasperich L-1617, Luxembourg City, Luxembourg; Academic Model Providing Access to Healthcare, P.O. Box 4606, Eldoret, 30100 Kenya; Assessorato Salute Regione Sicilia (Local Ministry of Health), Piazza Ottavio Ziino 24, Palermo, 90145 Italy

**Keywords:** Sea Migrants, Health status, Hospital referrals, Vulnerability, SORT IT, Operational Research

## Abstract

**Background:**

In recent years Europe has received an increasing influx of migrants, many of whom risked their lives crossing the Mediterranean Sea. In October 2013, Italy launched a search and rescue operation at sea in response to migrant deaths during the sea crossing. In August 2014, Médecins sans Frontières and the local Ministry of Health established an outpatient clinic at Augusta harbor, in Sicily, which received 26 % of total sea migrants arrived in Italy in 2014, to provide immediate medical assessment and care.

**Methods:**

This is a descriptive study of demographic and clinical data of sea migrants seen at the port clinic in Augusta from August to December 2014. We compared migrants from Near Eastern, war-torn regions (Group 1) and the others, mostly African (Group 2), as there were significant differences in terms of demographic and morbidity profiles.

**Results:**

There were 2593 migrants consulting the clinic (17 % af all rescued migrants) with 5 % being referred to hospital. Most were young males. The overall burden of vulnerability (pregnant women, children ≤5 years, unaccompanied minors, single parents with children of minor age, disabled and elderly persons) was 24 %. There were more small children, pregnant women, elderly, disabled, and persons with chronic diseases in Group 1, as compared to Group 2. Group 2 had more unaccompanied minors. Morbitidies in common were respiratory, dermatological, trauma-related and gastrointestinal conditions. However, acute and chronic cardiovascular disease, as well as diabetes, were more frequent in Group 1; chronic diseases affected 19 % of this group. Group 2 had more patients with skin diseases. Most migrants attributed their presenting symptoms to the perils of their journey. No risks for public health were detected.

**Conclusion:**

Among sea migrants, we identified two groups with different demographic and clinical characteristics, as well as vulnerability patterns. Overall morbidity suggested that the dangerous journey affected migrants’ health. Medical activities at reception sites should include screening for vulnerability and chronic disease management. Ensuring medical care to migrants on arrival can address European humanitarian obligations and provide support to local medical facilities.

## Background

Over the past 4 years, Europe has experienced an increasing influx of migrants crossing the Mediterranean Sea seeking humanitarian protection and/or improved living conditions [1, 2]. Most are fleeing war, persecution or extreme poverty [3].

In this study we define “migrants” as persons moving from one place to another, regardless of their legal status or reasons for travelling. All migrants in this study were rescued at sea, therefore we refer to them as sea migrants.

The number of sea migrants has escalated substantially since 2011 (62,500 in 2011, 43,000 in 2013, 170,000 in 2014) following the Syrian war, the rise of the *Islamic State*, as well as the political crisis in Libya. In 2015 about a million of them have reached Europe [1, 2].

Before 2011, the vast majority of sea migrants came from Africa (North and West Africa, and Horn of Africa), but recently, a significantly higher proportion of them has come from the Near East and Asia (Syria, Iraq, Afghanistan, Pakistan and Bangladesh) and includes migrants of minor age [1, 2, 4]. Furthermore, migration routes have been shifting, with Southern Italy being the major landing point up to 2014 and Greece representing the most crossed European border in 2015 [1, 2].

The sea journey, as well as the land crossing, are extremely dangerous and expose the migrants to risks that threaten their health [3, 5, 6]. Between October 2013 and November 2014 the Italian government implemented the search and rescue operation called *Mare Nostrum* (OMN) to address the increase in deaths of migrants during their sea crossing [3, 7]. The two main landing sites for rescued migrants were Augusta (Province of Siracusa) and Pozzallo (Ragusa), in Sicily [8, 9]. In 14 months, OMN rescued 160,000 sea migrants [10].

The extraordinary wave of sea migration in 2014 represented a challenge for Italian authorities in terms of reception capacity and providing adequate medical assistance. In addition, the Ebola epidemics in West Africa aroused fear of importation of communicable diseases that could represent a public health burden in Italy and the rest of Europe [11]. Therefore, following an official agreement with the local branch of the Ministry of Health (MoH), the medical humanitarian organization *Médecins Sans Frontières* (MSF) supported medical care for rescued migrants at both landing sites. The collaboration project in Augusta, the busiest port, started on August 1 and ended on December 31, 2014, when all activities were handed over to MoH.

There is some literature regarding sea migrants. Three previous studies analyzed medical activities in Malta in 2010–2011, in Lampedusa in 2010 as well as in 139 immigration centers operating in 13 Italian regions from May 2011 to June 2013. These studies showed that migrants were usually young men from African countries [12–14]. Although most were healthy or presented with minor health conditions, the main diseases observed were dermatological, respiratory and gastrointestinal. Furthermore, a significant part of their morbidity was related to the migration experience and/or the adjustment to the new environment in Italy. No major risks to public health were observed. Three previous studies focused on reasons for referral of migrants to hospitals in Lampedusa and Palermo [15–17].

In addition, studies in countries receiving a significant influx of migrants, including Italy, Spain, Germany and Greece, sought to describe health needs of immigrants as well as factors impairing their utilization of health care services [17–20]. However, these studies focused on the health status of migrants already settled in European countries.

What is lacking is accurate and up-to-date information regarding the burden of illnesses of migrants on their arrival in order to provide accessible and adequate medical care. As well, the changing profile of migrants has not been documented. This information is especially relevant for countries receiving the majority of them such as Greece, Italy and Germany during 2015. It is also important to document the health status of recently arrived migrants to properly evaluate their potential impact on European public health and health care systems.

The aim of this study was to define the demographic and clinical features, as well as referral patterns of newly arrived sea migrants seen at a MSF-MoH outpatient clinic at Augusta harbor from August 1–December 31, 2014. In addition, we compared migrants from Near Eastern war-torn regions (Syrians, Iraqis and Palestinians) versus those of all other nationalities (mostly from Africa), as there appeared to be substantial differences in terms of demographic and clinical characteristics.

## Methods

### Design

This is a descriptive study of routinely-collected project data.

### Setting

#### General setting

**Fig. 1:**
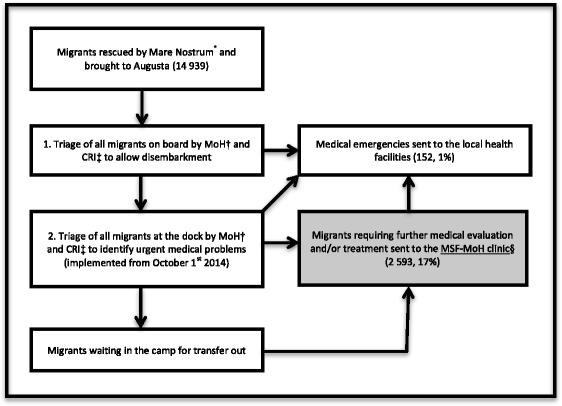
Flow of rescued sea migrants upon arrival at Augusta harbor (Italy) from Aug 1–Dec 31, 2014. *Search and rescue operation at sea launched by Italian Authorities in October 2013. †MoH - Ministry of Health. ‡CRI - Italian Red Cross. §MSF - Médecins sans frontières

In 2014 an unprecedented number of sea migrants arrived in Italy, with Sicily being the final destination for over 70 % of them [8, 9]. The death toll related to sea migration became a humanitarian challenge for the European Union [3, 5, 6]. In response, OMN was established by the Italian Government and Navy in October 2013 to actively search for and rescue migrants at sea [3, 7]. Rescue operations could involve several migrant boats. The largest military vessels offered medical first aid for rescued migrants and physicians of the MoH Prevention Department examined all migrants for communicable diseases on board before disembarkment (Fig. 1). Most rescued migrants were brought to Augusta (Province of Siracusa) and Pozzallo (Ragusa) as they are two large harbors on the eastern and southern coast of Sicily with easily accessible reception facilities for migrants [8, 9].

#### Specific Setting

Augusta, the busiest reception port, is located on the eastern Sicilian coast. Out of the 170,000 migrants who reached Italy by sea in 2014, 120,200 (71 %) landed in Sicily. Of these, 44,300 landed in Augusta (26 % of total migrants arriving in Italy) [8].

The MSF-MoH clinic was set up at Augusta harbor to provide medical care for migrants on arrival as well as to reduce overcrowding of local health facilities. It was located in the port camp and included tents for clinical evaluation and registration, observation and treatment (separated by gender), as well as a container used as a pharmacy.

#### Migrant flow

The migrant flow at Augusta port is shown in Fig. 1. They underwent one or two medical triages by MoH and Italian Red Cross (CRI): the first, on board the rescue vessel, aimed at identifying communicable diseases of public health significance; the second, on the dock, according to the CESIRA system, to briefly assess migrants’ health and select cases requiring hospital referral or treatment at the MSF-MoH clinic [21].

After triage, migrants reached the port camp where they were identified by Immigration Police, and subsequently transferred to temporary accommodation facilities outside the port. MSF-MoH medical activities were confined to the time from disembarkment to transfer out.

During this time, migrants with health complaints visited the clinic after being informed about services through promotional activities by MSF staff, NGOs or through other migrants. At times of lower workload, MSF engaged in active case-finding for migrants with medical issues and/or vulnerabilities.

#### Clinical activities

MSF staff, supported by MoH personnel, included two physicians, two nurses, and two to three cultural mediators. They spoke English, French, Arabic, Tigrinya, and Amharic, the most frequently spoken languages among the migrants.

The main MSF-MoH activities included medical consultations, clinical observation and treatment, organization of hospital referrals, follow-up of referred patients, communication with local MoH actors and NGOs, and pharmacy management. For each patient, a maximum of two presenting conditions were recorded. They were categorized by body systems or organs. We listed separately: trauma, the request for medical check-up and fever without infection signs, as they represented a significant burden of consultations. All presenting conditions not meeting the above definitions were categorized as “others”.

Diagnoses were made by physicians based on clinical assessment and simple tests (body temperature, blood pressure, blood sugar, urine dipstick, malaria rapid test, pregnancy test). Numbers and proportions for single diseases were not available since final diagnoses were not systematically recorded in the register. Due to time and context constrains we were unable to systematically screen for mental health conditions.

Screening for tuberculosis was based on patient interviews and physical examinations. Clinical suspects were sent to health facilities for testing. All treatments were provided according to national and MSF guidelines [22]. Due to potential contagiousness, patients with scabies were treated on site and recorded in a separate register.

A “health pass” containing demographic and clinical information was given to each patient with any clinical condition requiring medical follow-up, including chronic diseases, ongoing treatments, pregnant women, and children with incomplete or unknown vaccination status.

Criteria for referral to local health facilities were based on severity of illness, presence of vulnerability, and the need for advanced diagnostics and/or specific treatment.

### Study population

This study included all patients seen at the MSF-MoH clinic from August 1–December 31, 2014. Patients were sent to the clinic upon triage at the dock or consulted the clinic spontaneously, or upon active case-finding. Whenever possible, MSF staff tried to identify and examine patients with vulnerabilities (defined as pregnant women, children ≤5 years, unaccompanied minors, single parents with children of minor age, disabled and elderly persons). Age groups and vulnerabilities were determined according to their clinical relevance in the specific setting and based on MSF conventions.

### Data sources, data collection and variables

Information about each landing, including total number of migrants, number of adult males and females, number of accompanied and unaccompanied minors, and nationality was provided by Italian Authorities (Immigration Police). Information about the origin of the boat(s), the number of days spent at sea, possible adverse events during the sea crossing, such as shipwreck, was obtained directly from the migrants.

Patient data were collected by MSF staff during each consultation as well as through follow-up of hospital-referred cases, and entered manually onto paper registers, followed by single-entry into an Excel database (Microsoft Excel, 2011). All fields in the database were validated through crosschecking with paper data.

### Analysis and statistics

Categorical variables were summarized using frequencies and proportions; medians and interquartile range were reported for skewed continuous variables. Comparisons of proportions were done using the Chi-square test where appropriate, or alternatively the Fisher’s Exact test, with levels of significance set at 5 %. Only significant *p*-values (*P* < 0.05) were recorded in the tables. Sample size calculation was not required as all patients were included.

### Ethics approval and consent to participate

Consent for utilization of data for analysis and publication was obtained from local MoH and Italian MSF Coordination. Local ethics approval was obtained from the Italian Ministry of Health.

The study met the MSF Ethics review Board (Geneva, Switzerland) approved criteria for studies of routinely collected data and was also approved by the Ethics Advisory Group of the International Union Against Tuberculosis and Lung Disease, Paris, France. Informed consent was not sought from research subjects, as the study was conducted as a retrospective analysis of routine programme data. Anonymity and confidentiality were maintained, and the research subjects were assured the same complement of health services as mandated by the MoH.

## Results

During the study period, OMN performed 51 landings at the Augusta harbor, rescuing a total of 14,939 migrants. Most migrants’ boats came from Libya (32 rescue events) and Turkey (15) but others came from Egypt and Syria. Boats from different origins could have been rescued in the same event. In six rescue events (12 %), migrants reported having risked their lives during the sea journey due to a shipwreck and/or incidents during rescue operations.

Table 1 shows details about the landings and time in the port. In 61 % of landings, migrants stayed at the harbor less than 24 h. In only two landings was this period greater than 96 h.

**Table 1:** Characteristics of landings at Augusta harbor (Italy) from Aug 1–Dec 31, 2014

Variable	TOTAL	
	n	(%)
No. landings	51	(100)
No. of migrants per landing		
< 200	24	(47)
201–400	13	(25)
401–600	11	(22)
> 600	3	(6)
*Median*	*225*	
*(IQR)*	*(152–414 · 5)*	
Time to transfer out^a^ (hours)		
< 24	31	(61)
24–48	18	(35)
49–96	0	(0)
> 96	2	(4)
*Median*	*12*	
*(IQR)*	*(6–29 · 5)*	
Reported no. days spent at sea^b^		
Total Range	18 *·* 5–1	
*Median (IQR)*	*4 (2 · 5–9)*	

^**a**^Time between disembarkment and transfer out of the port

^b^Information obtained during patient interviews

Demographic characteristics of migrants at time of landing are shown in Table 2. The majority of adults were males and 17 % were minors, of whom 25 % were unaccompanied.

**Table 2:** Demographic characteristics of rescued sea migrants landed at Augusta harbor (Italy) from Aug 1–Dec 31, 2014

Variable	n	(%)
No. migrants landed	14,939	(100)
Age category		
Total	14,939	(100)
Adults (≥18 years)	12,361	(83)
Minors (<18 years)	2578	(17)
Adults (≥18 years)		
Total	12,361	(100)
Male	10,824	(88)
Female	1537	(12)
Minors (<18 years)^a^		
Total	2578	(100)
Accompanied	1929	(75)
Unaccompanied	649	(25)

^a^Gender distribution for minors was not always recorded by Immigration Police

Of 14,939 migrants landed at Augusta during the study period, 2593 (17 %) consulted the MSF-MOH clinic and 152 (5 % of clinic patients and 1 % of all migrants landed) were referred to hospital; 19 migrants refused referral.

**Fig. 2:**
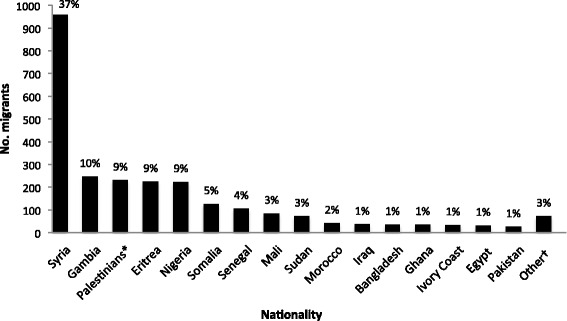
Nationality of rescued sea migrants seen at the MSF-MoH clinic at Augusta harbor (Italy) from Aug 1–Dec 31, 2014. *Palestinians living in Gaza, Syria, Lebanon and Libya. †Nationalities with < 1 % frequency: Algeria, Benin, Burkina Faso, Cameroun, Central African Republic, Chad, Democratic Republic of Congo, Ethiopia, Guinea Bissau, Guinea Conakry, Libya, Niger, Sierra Leone, Togo, Tunisia, Yemen

The largest number of migrants consulting at the clinic came from Syria. Other nationalities are shown in Fig. 2.

Demographic features and the vulnerability profile of sea migrants seen at the clinic overall and stratified by region of origin are reported in Table 3. Migrants were predominantly men between 18 and 30 years old and 24 % presented with vulnerabilities, which include pregnant women, children ≤5 years, elderly persons (≥60 years), unaccompanied minors, single parents with children of minor age and persons with disabilities. There were significantly more women, children ≤5 years and older adults (>30 years) in Group 1 (Syrian, Iraqi and Palestinian migrants) than in Group 2 (all other nationalities). Migrants from Group 2 were mainly 6–30 years old. Group 1 presented with significantly more vulnerabilities than Group 2 including being ≤5 years old, ≥60 years old, pregnant and disabled. Unaccompanied minors were the most frequent vulnerability in Group 2.

**Table 3:** Demographic features and vulnerability profile of rescued sea migrants seen at the MSF-MoH clinic at Augusta harbor (Italy) from Aug 1–Dec 31, 2014, stratified by region of origin

Variable	TOTAL		Syria, Palestine, Iraq		Others^a^		*P-value* ^*b*^
	n	(%)	n	(%)	n	(%)	
Sex							
Total	2593	(100)	1227	(100)	1366	(100)	
Males	2101	(81)	906	(74)	1195	(87)	*<0.001*
Females	492	(19)	321	(26)	171	(13)	
Age, years							
Total	2593	(100)	1227	(100)	1366	(100)	
≤ 5	218	(8)	195	(16)	23	(2)	*<0.001*
6–17	344	(13)	120	(10)	224	(16)	*<0.001*
18–30	1373	(53)	444	(36)	929	(68)	*<0.001*
31–59	589	(23)	415	(34)	174	(13)	*<0.001*
≥ 60	47	(2)	46	(4)	1	(<1)	*<0.001*
Not recorded	22	(1)	7	(<1)	15	(1)	
Presence of vulnerability (definitions below)							
Total	2593	(100)	1227	(100)	1366	(100)	
Yes	612	(24)	332	(27)	280	(20)	*<0.001*
No/Not recorded	1981	(76)	895	(73)	1086	(80)	
Burden of vulnerabilities							
Total	619^c^	(100)	337^c^	(100)	282^c^	(100)	
≤ 5 years old	218	(35)	195	(58)	23	(8)	*<0.001*
Unaccompanied minor	201	(32)	0	(<1)	201	(71)	*<0.001*
Pregnant	69	(11)	42	(12)	27	(10)	*<0.001*
Disabled	69	(11)	48	(14)	21	(7)	*<0.001*
≥ 60 years old	47	(8)	46	(14)	1	(<1)	*<0.001*
SPCMA^d^	15	(2)	6	(2)	9	(3)	

^**a**^All other nationalities

^b^Significant *P*-values only (<0.05) are shown in the table

^c^6 patients presented with two vulnerabilities

^d^SPCMA—Single parent with children of minor age

Thirty-three percent of children ≤5 years had an incomplete or unknown vaccination status.

Table 4 shows the clinical characteristics of the migrants. Of those screened for tuberculosis, 10 (<1 %) were non-confirmed clinical suspects and one suspect was subsequently confirmed at the hospital. Two migrants reported to have been previously diagnosed with tuberculosis and were already on treatment.

**Table 4:** Clinical features of rescued sea migrants seen at the MSF-MoH clinic at Augusta harbor (Italy) from Aug 1–Dec 31, 2014, stratified by region of origin

Variable	TOTAL		Syria, Palestine, Iraq		Others^a^		*P-value* ^*b*^
	n	(%)	n	(%)	n	(%)	
Outcome of tuberculosis screening							
Total	2593	(100)	1227	(100)	1366	(100)	
No clinical suspicion	2573	(99)	1225	(99)	1348	(99)	*<0.001*
Clinical suspicion, not confirmed	10	(<1)	1	(<1)	9	(<1)	*0.02*
Clinical suspicion, confirmed	1	(<1)	0	(0)	1	(<1)	
Previously confirmed and already on therapy	2	(<1)	0	(0)	2	(<1)	
Not recorded	7	(<1)	1	(<1)	6	(<1)	
Presenting conditions							
Total	2987^c^	(100)	1393^c^	(100)	1594^c^	(100)	
Respiratory	629	(21)	320	(23)	309	(19)	*0.02*
Dermatological	585	(20)	159	(11)	426	(27)	*<0.001*
Trauma	344	(12)	152	(11)	192	(12)	
Gastrointestinal	299	(10)	157	(11)	142	(9)	*0.03*
Neurological	233	(8)	94	(7)	139	(9)	*0.05*
Medical check-up^d^	125	(4)	107	(8)	18	(1)	*<0.001*
Dental	104	(3)	48	(3)	56	(4)	
Ophthalmological	98	(3)	40	(3)	58	(4)	
Urogenital	79	(3)	35	(3)	44	(3)	
Cardiovascular incl. hypertension	53	(2)	49	(4)	4	(<1)	*<0.001*
Fever	52	(2)	25	(2)	27	(2)	
Gyneco-obstetric	51	(2)	31	(2)	20	(1)	*0.04*
Endocrinological	25	(1)	20	(1)	5	(<1)	*<0.001*
Mental-health related	17	(1)	10	(1)	7	(<1)	
Other^e^	290	(10)	145	(10)	145	(9)	
Not recorded	3	(<1)	1	(<1)	2	(<1)	
Symptoms’ onset							
Total	2593	(100)	1227	(100)	1366	(100)	
Before migration	373	(14)	216	(18)	157	(11)	*<0.001*
During migration	1873	(72)	761	(62)	1112	(81)	*<0.001*
Since arrival	218	(8)	140	(11)	78	(6)	*<0.001*
Does not apply^f^	127	(5)	108	(9)	19	(1)	*<0.001*
Not recorded	2	(<1)	2	(<1)	0	(0)	
Presence of chronic disease (definitions below)							
Total	2593	(100)	1227	(100)	1366	(100)	
Yes	291	(11)	232	(19)	59	(4)	*<0.001*
No/Not recorded	2302	(89)	995	(81)	1307	(96)	
Burden of chronic disease							
Total	313^g^	(100)	254^g^	(100)	59^g^	(100)	
Cardiovascular	86	(27)	81	(32)	5	(8)	*<0.001*
Diabetes	53	(17)	48	(19)	5	(8)	*0.05*
Orthopedic	33	(11)	27	(11)	6	(10)	
Gastrointestinal	30	(10)	21	(8)	9	(15)	
Chronic lung disease	18	(6)	14	(6)	4	(7)	
Neurological	15	(5)	12	(5)	3	(5)	
Urogenital	10	(3)	5	(2)	5	(8)	*0.01*
Dermatological	3	(1)	3	(1)	0	(0)	
Endocrinological^h^	3	(1)	3	(1)	0	(0)	
Other^i^	62	(20)	40	(16)	22	(37)	*<0.001*

^a^All other nationalities

^b^Significant *P*-values only (<0.05) are shown in the table

^c^For each patient we could record a maximum of two clinical conditions (16 % of patients seen)

^d^Patients consulting mostly for blood pressure, blood sugar or temperature check

^e^Most common: fatigue, discomfort, pain (myalgia, arthralgia, skeletal pain, etc.), otitis/otalgia, abscess, dizziness, dehydration, agitation and panic attack, anemia, rheumatic disease, need for chronic medications, loss of eyeglasses, malnutrition, palpitation, hearing loss

^f^For patients coming for check-up

^g^22 patients (all from the group “Syria, Palestine, Iraq”) presented with 2 chronic diseases, mainly cardiovascular disease and diabetes

^h^Hypothyroidism and hyperthyroidism

^i^Most common: congenital diseases (e.g. anemia), autoimmune diseases, disabilities, mental illnesses, nephrolithiasis, renal failure, chronic ear or eye disease, prostatic hypertrophy

The most frequent presenting conditions involved the respiratory tract, followed by skin and trauma-related conditions.

A significantly higher number of Group 1 migrants consulted because of cardiovascular, endocrinological (diabetes), respiratory, gastrointestinal and gyneco-obstetric problems, while skin and neurological conditions (almost exclusively headache) were more frequent in Group 2. We diagnosed 354 scabies cases, 95 % of which belonged to Group 2 (25 % prevalence in Group 2).

Additionally, Group 1 migrants consulted at the clinic for a medical check-up more often than Group 2 migrants.

Overall, most of the study population reported symptoms’ onset *during migration*. However, more migrants from Group 1 reported symptoms’ onset *before migration* as well as *since landing* as compared to Group 2.

The prevalence of chronic disease was significantly higher in Group 1, including cardiovascular disease and diabetes, which were the most common co-morbidities.

Table 5 shows presenting conditions in detail.

**Table 5:** Most common presenting conditions at the MSF-MoH clinic at Augusta harbor (Italy) from Aug 1–Dec 31, 2014, by category

Category	Diagnoses
Respiratory	Asthma, chronic obstructive lung disease, upper and lower respiratory tract infection
Dermatological	Burn, head and pubic lice, psoriasis, skin infection (including scabies), skin rash (including allergy), wound
Trauma	Contusion, fracture, joint dislocation, pain and/or functional disability following physical trauma or due to presence of foreign body
Gastrointestinal	Abdominal and epigastric pain, gastroenteritis, hemorrhoids, nausea, perianal abscess and fissure
Neurological	Headache (almost exclusively), known brain tumor, seizure
Check-up	Blood pressure, blood sugar or temperature check
Dental	Dental abscess, tooth pain
Ophthalmological	Cataract, conjunctivitis, decreased sight
Urogenital	Nephrolithiasis, urinary tract and genital infections
Cardiovascular including hypertension	Chest pain, collapse, hypertensive crisis, hypotension
Fever	Fever with no infection signs
Gyneco-obstetrical	Abdominal pain in pregnant woman, absence of fetal movements, suspected pregnancy, vaginal bleeding in pregnant woman
Endocrinological	Diabetes (almost exclusively), hypoglycemia
Mental-health related	Depression, schizophrenia
Other	Abscess, agitation and panic attack, anemia, dehydration, discomfort (non-specific), dizziness, fatigue, hearing loss, loss of eyeglasses, malnutrition, need for drug supply, otitis/otalgia, pain (myalgia, arthralgia, rachialgia, etc.), palpitations, rheumatic disease

Table 6 shows the number of patients referred to the hospital, their vulnerabilities as well as reasons and outcomes of referral. Of those referred, 20 % were hospitalized. Nearly half of migrants referred to hospital presented with vulnerabilities, the most frequent being pregnancy. The three most common reasons for referral were related to trauma, gyneco-obstetric and respiratory conditions. There were no significant differences between the two groups in terms of frequency, outcomes and reasons of referral.

**Table 6:** Outcomes and reasons for hospital referrals from Augusta harbor (Italy) from Aug 1–Dec 31, 2014

Variable	n	(%)
No. patients referred	152^a^	(100)
Presence of vulnerability (definitions below)		
Yes	70	(46)
No/Not recorded	82	(54)
Burden of vulnerabilities		
Total	70	(100)
Pregnant	37	(53)
≤ 5 years	10	(14)
Unaccompanied minor	9	(13)
Disabled	8	(11)
≥ 60 years	5	(7)
SPCMA^b^	1	(1)
Reasons for referral		
Total	152	(100)
Trauma	35	(23)
Gyneco-obstetrical	33	(22)
Respiratory	20	(13)
Cardiovascular incl. hypertension	11	(7)
Gastrointestinal	10	(7)
Neurological	7	(5)
Dermatological	6	(4)
Surgical	5	(3)
Fever	5	(3)
Urogenital	4	(3)
Medical check-up	3	(2)
Endocrinological	2	(1)
Dental	1	(<1)
Mental-health related	1	(<1)
Other^c^	9	(6)
Outcome of referral		
Total	152	(100)
Discharged from the Emergency Room	110	(72)
Hospitalized	31	(20)
Not recorded	9	(6)
Left before diagnosis/treatment completed	2	(2)

^a^12 Patients were referred directly to hospital from triage and did not consult the MSF-MoH clinic, therefore hospitalization rate for clinic patients was 5 % (1 % overall)

^b^SPCMA—Single parent with children of minor age

^c^Collapse, dehydration, otitis, hypothermia, malnutrition

## Discussion

This is the first study assessing the demographic and clinical features of sea migrants at the time of arrival to Europe. This information has never been more important, considering the challenge many European countries are currently facing of ensuring quality medical care to newly-arrived migrants.

A major finding was that there were two migrant groups that differed significantly in terms of demographic and clinical characteristics, as well as patterns of vulnerability. Migrants from the Near East (Syrians, Iraqis and Palestinians) included a large proportion of small children, pregnant women, elderly and disabled people. Among this group there was more acute and chronic vascular disease, diabetes, and health-seeking behavior. The other group included mostly African migrants of young age. Among them there were more unaccompanied minors and patients with skin diseases, including scabies.

However, some diseases of the two groups were similar, with the most common being respiratory, dermatological and gastrointestinal conditions, as well as trauma. We suggest these are likely associated with the dangerous journey. Migrants travelled for days on unseaworthy and overcrowded boats, where hygiene conditions were poor, the climate was cold and wet, water and food were scarce and often contaminated [3, 5, 23]. Injuries could be sustained while getting onto the migrant boat or during rescue operations. Some suffered immersion in the sea. Prior to the sea crossing, many had undertaken long journeys across the desert followed by difficult living conditions in Libya, with repeated violence and exploitation. This contributed to the burden of trauma-related conditions [24].

A positive feature of this project was that the MSF-MoH clinic appeared to relieve local hospital facilities of some of the burden of care. Only 5 % of clinic patients and 1 % of all landed migrants were sent to hospital. There was efficient cooperation between MSF and MoH clinical staff and good relations with the local hospitals. As a result of the collaboration, MoH took over medical activities at the Augusta harbor in January 2015.

Like ours, earlier studies showed that African migrants were young men and had respiratory and gastrointestinal morbidities as well as traumatic injuries [12–14, 17].

Among all studies, pregnancy-associated conditions were the most common reasons for hospital referral among women [12, 13, 15, 16].

Previous studies of sea migrants did not include the presence of large numbers of migrants from Near Eastern, war-torn regions, who often suffer from chronic diseases and who have a higher and different burden of vulnerabilities. However, our findings are supported by more recent studies conducted with Syrian refugees in Jordan and Lebanon, which focused on the burden of non-communicable diseases [25, 26].

A number of European studies have focused on communicable diseases in migrants with contradictory findings [14, 27–29]. Our study showed a low burden of infectious diseases of public health significance. There were no suspected cases of Ebola among migrants from Africa, as would be expected following a journey through the Sahara desert that was much longer than the incubation period of the virus.

We note that this study was a snap shot of migrants at a particular time and place. Since then, the migration routes have been changing and further assessments of migrants’ health in different contexts will be required to provide adequate medical care.

There are a number of policy implications from this study: 1) It is important to have adequate and accessible medical services at landings to provide immediate care to newly-arrived migrants. They can relieve the burden on local health care facilities and coordinate referrals; 2) Medical care needs to be organized to manage both acute and chronic diseases, given the different profiles of migrants arriving; 3) Care for migrants should be sensitive to screen for and manage vulnerabilities. Vaccination status of children should be systematically assessed and vaccines readily available; 4) Adequate training should be offered to health professionals dealing with migrants’ health, since this requires not only medical and organizational skills, but also ethical and legal expertise, as well as cultural sensitivity; 5) Comprehensive and accurate data collection of migrants’ health needs should be established to properly inform relevant authorities in Europe.

The strengths of this study include a large sample size that was representative of the sea migrants landed during this time period in Italy in terms of demographics and countries of origin. Data collection and entry were validated through cross-checks. The study adhered to the STROBE guidelines [30].

### Limitations

There were some limitations in this study. Although it provides useful insights into migrants’ health care, the relatively small study population and narrow time of the intervention could not capture the full picture of the migration issue in all its medical and public health implications. The clinic was temporary and diagnostic testing was very limited, hence the lack of disease specificity. The rapid turnover of migrants limited follow-up of referred patients and prevented assessment of mental health status, which would be important given the traumatic journey. Finally, the study population mostly represented migrants who were actively seeking medical attention, which may be source of selection bias.

## Conclusions

This study shows that there were different profiles of migrants landing in Southern Italy during 2014, having diverse medical requirements. Providing adequate initial medical care to them can address humanitarian obligations and provide support to local medical facilities. These services should be part of the European Union’s response to the migrant crisis. Systematic data collection in similar contexts is needed to improve knowledge and training of health care professionals, as well as the level of care for migrants.

## Abbreviations

CESIRA: Coscienza, Emorragia, Shock, Insufficienza respiratoria, Rottura ossea, Altro
CRI: Croce Rossa Italiana (Italian Red Cross)
MoH: Ministry of Health
MSF: Médecins sans frontières
NGO: Non-Governmental Organization
OMN: Operation *Mare Nostrum*
SPCMA: Single Parent with Children of Minor age
STROBE: STrengthening the Reporting of OBservational studies in Epidemiology
